# Ninjin’yoeito Improves Genitourinary Symptoms in Patients With Frailty

**DOI:** 10.7759/cureus.40767

**Published:** 2023-06-21

**Authors:** Nobuo Okui, Machiko.Aurora Okui

**Affiliations:** 1 Dentistry, Kanagawa Dental University, Yokosuka, JPN; 2 Urogynecology, Dr. Okui’s Urogynecology and Urology Clinic, Yokosuka, JPN

**Keywords:** older female, older adult, integrative/complementary medicine, polypharmacy, elderly female, frailty syndrome, frailty, genitourinary syndrome of menopause, overactive bladder, ninjin'yoeito

## Abstract

Introduction

In geriatric medicine, there is currently significant attention on frailty, a condition commonly associated with aging and characterized by muscle weakness and other age-related changes. Within the fields of urology and gynecology, conditions such as overactive bladder (OAB) and genitourinary syndrome of menopause (GSM) have been identified as crucial concerns due to their negative impact on the quality of life of elderly individuals. In this study, we investigated the potential of Ninjin'yoeito (NYT), a traditional Chinese herbal medicine, as a viable treatment option for frailty. Additionally, we hypothesized that NYT may also contribute to the improvement of symptoms associated with OAB and GSM, and potentially help in reducing the dosage of OAB medications.

Methods

In this retrospective cohort study conducted from November 2016 to November 2022, we created a website describing the relationship between frailty and genitourinary symptoms in frail patients aged ≥ 65 years with GSM who underwent pelvic floor muscle training. The patients were divided into two propensity score-matched groups: NYT group (received NYT for one year) and no-NYT group (did not receive NYT), based on their wishes. The fatigue, resistance, ambulation, illnesses, and loss of weight (FRAIL) scale was used to assess frailty status. Urinary symptoms were evaluated using the International Consultation on Incontinence Questionnaire-Short Form (ICIQ-SF) and Overactive Bladder Symptom Score (OABSS). Genital symptoms were investigated using the vaginal health index score and vulvodynia swab test. The value of each score was obtained before (T0) and 12 months after (T12) treatment, and the difference (Δ_T0/T12_) was calculated.

Results

During the study period, 985 outpatients visited our clinic, of whom 725 were considered frail/pre-frail; 402 women with frailty/pre-frailty (mean age 77.5 ± 6.49 years) were included, with a median follow-up of 14.5 months. The NYT and no-NYT groups had 220 and 182 patients, respectively. After propensity score matching, each group had 159 patients. Δ_T0/T12_FRAIL scale score was significantly higher in the NYT group (0.13 ± 0.37) than in the no-NYT (0.01 ± 0.10) group (p=0.001.) However, urinary symptoms improved in the NYT group more than in the no-NYT in terms of the following parameters: Δ_T0/T12_OABSS (NYT: 0.89 ± 1.65; no-NYTl: 0.36 ± 1.14, p=0.001) and Δ_T0/T12_ICIQ-SF score (NYT: 1.51 ± 1.75; no-NYT: 0.42 ± 1.18, p<0.001). Genital symptoms were better in the NYT group in terms of _ΔT0/T12_VHIS (NYT: 0.58 ±1.08; no-NYT: 0.21 ±0.65, p<0.001). The vulvodynia swab test showed improvements in left para-hymen evoked pain in both groups. In the NYT group, 5% of the patients underwent antimuscarinic drug dose reduction for overactive bladder treatment. NYT use was not associated with significant side effects, and only 0.6% of patients reported drug allergies.

Conclusion

NYT improved activity levels in frail/pre-frail patients. Moreover, NYT use improved various genitourinary symptoms experienced by patients with frailty/pre-frailty. Treatment with NYT may reduce the dose of overactive bladder medications. The anticholinergic load-reducing effect of NYT may help solve the problem of polypharmacy.

## Introduction

Geriatric medicine is currently receiving significant attention [1]. Frailty, in particular, is a crucial concern within geriatric medicine [2]. Frailty is a clinical condition associated with aging, characterized by muscle weakness and heightened susceptibility to internal and external stressors [3,4]. In the field of geriatric medicine, specifically urology, and gynecology, conditions such as overactive bladder (OAB) [5] and genitourinary syndrome of menopause (GSM) have been identified as having detrimental effects on the quality of life for older patients [6,7]. Similar to frailty, OAB and GSM stem from hormonal changes that lead to tissue frailty and significantly impact overall well-being [3,7]. The risk factors for frailty include urological and gynecological issues, physical inactivity, cognitive impairment, malnutrition, and polypharmacy. From a social medicine perspective in geriatrics, polypharmacy intersects with these conditions [8]. Polypharmacy, especially in frail, older individuals, can result in drug interactions, and the failure to take medications or forgetting doses can hinder proper medication use, leading to various serious health problems [8]. There is also a connection between urology and social medicine in this context, as medications used for OAB often have notable anticholinergic effects [5,9].

Clinical experience in Japan suggests that Ninjin'yoeito (NYT), a traditional Chinese herbal medicine, may alleviate symptoms of frailty and OAB in older patients, although this has not been previously reported [10-13]. We hypothesized that NYT use can improve several genitourinary symptoms in patients with frailty. In this study, we aimed to determine whether the combination of pelvic floor muscle training (PFMT) and NYT is associated with improved genitourinary symptoms when compared to PFMT alone in older women with frailty.

## Materials and methods

Study design and approval 

This single-center, retrospective, propensity score (PS)-matched [12] cohort study was approved by the Kanagawa Association of Medical and Dental Practitioners (approval number: 22-001). The study was conducted at Yokosuka Urogynecology and Urology Clinic, Yokosuka, Kanagawa, Japan, and was registered at the University Hospital Medical Information Network Clinical Trials Registry (UMIN-CTR) (clinical study number: R000057695). Data were extracted from the electronic medical records of patients at our institution.

Patients

All patients (women aged at least 65 years) were recruited via the Internet and underwent PFMT. We created a website describing urinary symptoms, genital symptoms, and frailty that was active from November 2016 to November 2022. We advertised the treatment on the website using keywords such as "PFMT," "frequent urination," "urinary incontinence," "overactive bladder," and "external genital pain." Patients who requested treatment took an online appointment for treatment. Patients themselves filled out the fatigue, resistance, ambulation, illnesses, and loss of weight (FRAIL) scale, International Consultation on Incontinence Questionnaire-Short Form (ICIQ-SF) [14], and OAB symptom score (OABSS) [15] questionnaires in the presence of a nurse. Vaginal health index score (VHIS) evaluation and vulvodynia swab test were performed by a physician.

T0 was defined as the time of first visit to our hospital by patients who had been evaluated for OAB, were on OAB medication, or had no OAB. For patients who had not been evaluated for OAB, T0 was considered to be one month after evaluation and appropriate OAB medication initiation; the patients underwent the same evaluations after T0.

The FRAIL score at T0 was used to assess frailty status. NYT was prescribed to patients with frailty and pre-frailty, based on their desire. All patients were evaluated every three months; a pharmacist checked the leftover medications during each follow-up evaluation. Medication use was continued for at least 12 months, and patients were evaluated at 12 months, which corresponded to T12. Patients were divided into two groups, based on their treatment desires: NYT (received NYT for 12 months) and no-NYT (did not receive NYT) groups.

All patients provided informed consent. We included patients aged at least 65 years, with American Society of Anesthesiologists (ASA) physical status class I-III. Patients with follow-up < 1 year and those with pelvic organ prolapse (POP) stage 2 or higher were excluded.

Method of evaluation

Using the FRAIL questionnaire, the patients were interviewed on the following five items: fatigue, resistance, ambulation, illnesses, and loss of weight. Each item was rated on a 1-point scale; scores of 1-2 or ≥ 3 out of 5 were considered to represent pre-frailty and frailty, respectively. The ICIQ-SF questionnaire evaluates three aspects of urinary incontinence: frequency, volume, and impact on daily life. Mild, moderate, and severe incontinence were indicated by scores of 1-5, 6-12, 13-18, and 19-20, respectively. The OABSS questionnaire assesses the severity of OAB using four items: daytime urinary frequency, nighttime urinary frequency, urinary urgency, and urinary incontinence. The score ranges from 0 to 15, with <5, 6-11, and ≥12 points connoting mild, moderate, and severe OAB, respectively.

The following items were used to evaluate the VHIS: overall elasticity, fluid secretory characteristics, vaginal pH, epithelial mucosa, and moisture. These five items were rated on a scale of 1 (very poor) to 5 (very good). A total VHIS score <15 indicated poor vaginal health. The vulvodynia swab test was used to determine localized or generalized induced vulvodynia. It involves the assessment of the degree and characteristics of pain by applying pressure to various areas such as the para-hymen, urethra, and labia.

PFMT

During each visit, a nurse and physician provided PFMT. The physician instructed the patient to squeeze the vagina in a particular direction using a finger placed intravaginally. A video was created and uploaded on YouTube to help patients perform PFMT at home. Patients performed PFMT at home for 30 minutes daily. During each visit, we reviewed the patient’s daily logbook containing the daily PFMT exercises and encouraged patients who performed the exercises.

NYT exposure

Patients were administered 7.5 g/day of NYT granules (KB-108, Kracie Pharma Ltd., Tokyo, Japan). This drug was covered by insurance, and the dose used was in accordance with the Japanese Pharmaceuticals and Medical Devices Agency recommendations [13]. The pharmacist verified medication adherence by quantifying the leftover medication, which was returned to the pharmacy. Patients with medication discontinuation were considered to have participated in the study for < 1 year, and therefore were excluded from the study. In the case of an adverse event, the NYT package information and medical opinion were used to determine a causal relationship between NYT use and the adverse effect.

OAB treatment drug

To facilitate statistical analysis, OAB medication use was defined as using ≥50% of the prescribed OAB medications within three months before the interview, regardless of the amount of medications taken. Use of <50% of the prescribed OAB medications within three months before the interview was considered medication non-use. The antimuscarinic drugs included solifenacin succinate, oxybutynin hydrochloride, propiverine hydrochloride, tolterodine, imidafenacin, and fesoterodine fumarate. The β3 adrenoceptor agonists were mirabegron and vibegron. These drugs were covered by insurance, and the doses used were in accordance with the Japanese Pharmaceuticals and Medical Devices Agency recommendations [13].

The pharmacist confirmed patient adherence to the OAB medication by quantifying the amount of leftover medication returned to the pharmacy. The physician determined the reason for medication discontinuation, which was used to divide the patients into two categories: Category A consisted of patients who were no longer eligible for OAB medication use due to the effects of NYT and PFMT. We considered this category as the therapeutic effect group and performed a statistical analysis of the rate of decrease in OAB medication use. Category B patients were those who forgot to take the medication or did not want to take it due to side effects. These patients were considered to have participated in the study for < 1 year, and hence were excluded from the study.

Statistical analysis

Student's t-test was used to compare continuous variables between the two groups. Statistical significance was set at p < 0.01. The differences in parameters between T0 and T12 (Δ_T0/T12_) were calculated. Fisher’s exact test was used for between group comparison of discrete variables. The summary statistics are expressed as mean (standard deviation) or number (percentage). All analyses were performed using R version 2.15.1 (Released 2012; R Core Team, Vienna, Austria) and the EZR package. All statistical analyses were created and validated using Python code (Python Software Foundation, Wilmington, Delaware, United States) with ChatGPT3.5 (OpenAI, San Francisco, California, United States). All operations were conducted on the Windows 10 version of the 1903 operating system (Microsoft Corporation, Redmond, Washington, United States).

## Results

**Figure 1:**
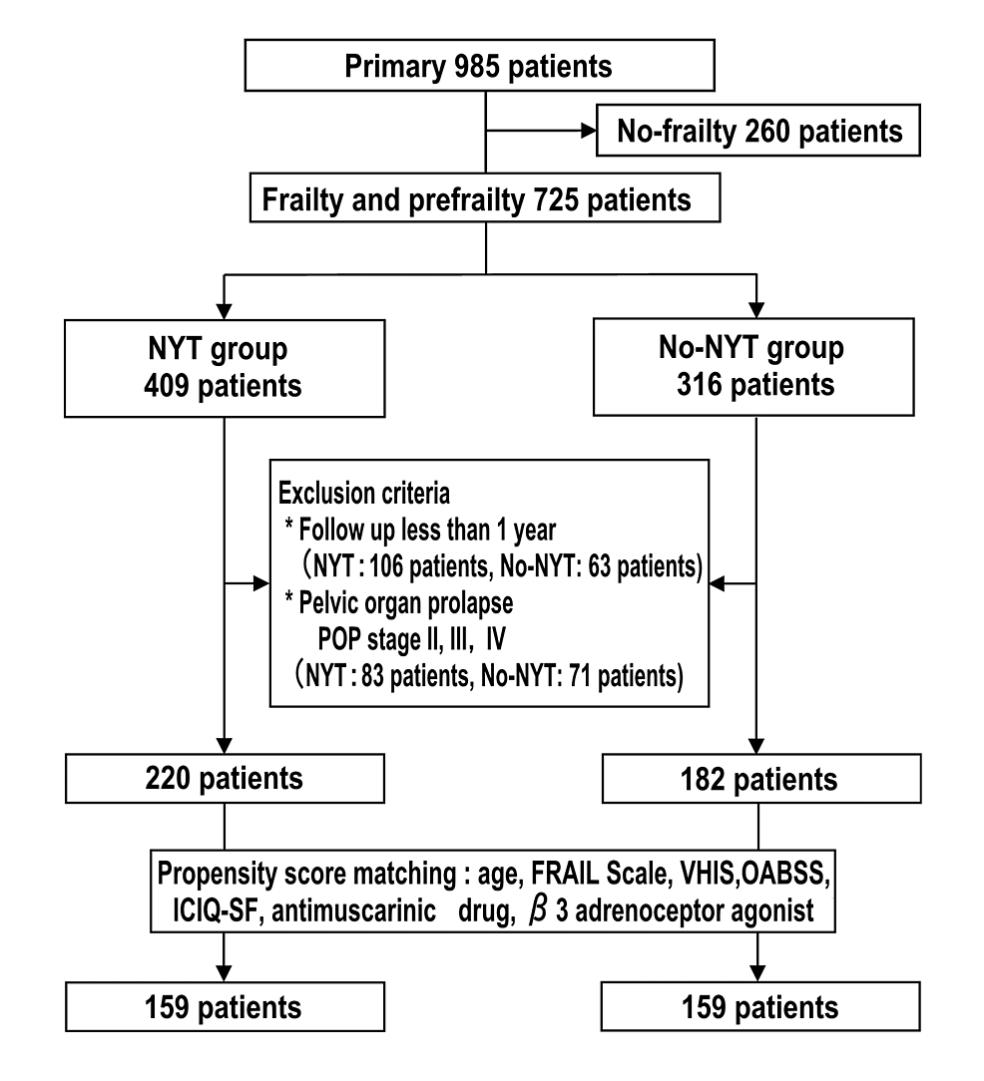
Study flowchart showing the inclusion and exclusion criteria Patients in the NYT group received NYT for one year, whereas those in the no-NYT group did not receive NYT. POP, Pelvic organ prolapse; ICIQ-SF, International Consultation on Incontinence Questionnaire-Short Form; OABSS, overactive bladder symptom score; VHIS, vaginal health index score; NYT, Ninjin'yoeito; FRAIL, fatigue, resistance, ambulation, illnesses, and loss of weight

Of 985 outpatients who were consulted in our clinic during the study period, 725 and 260 were frail/pre-frail and non-frail, respectively, based on the FRAIL score. Of the 725 frail/pre-frail women, 402 (mean age 77.5 ± 6.49 years) met the eligibility criteria, with a median follow-up of 14.5 months. The NYT and No-NYT groups had 220 and 182 women, respectively; after PS matching, each group had 159 women (Figure 1).

Table 1 shows participant characteristics. The two groups were compared in terms of angina, arthritis, asthma, cancer, chronic lung disease, congestive heart failure, diabetes, heart attack, kidney disease, and stroke, using the illness subscale of the FRAIL scale. There were no significant between group differences after PS matching.

**Table 1:** Between group comparison after PS matching BMI, body mass index; NYT, Ninjin'yoeito; PS, propensity score; FRAIL: fatigue, resistance, ambulation, illnesses, and loss of weight

	NYT group (n=159)	No-NYT group (n=159)	p-value
Age	78.13 ±5.93	78.03±5.76	0.878
BMI	22.61 ± 2.83	22.70 ± 2.96	0.427
FRAIL Scale	1.87 ±1.35	1.73±1.27	0.325
Angina	19 (11.9％)	19 (11.9％)	1.000
Arthritis	19 (11.9％)	23 (14.5％)	0.500
Asthma	14 ( 8.8％)	17 (10.7％)	0.706
Cancer	45 (28.3％)	43 (27.0％)	0.900
Chronic lung disease	10 ( 6.3％)	7 ( 4.4％)	0.619
Congestive heart failure	40 (25.2%)	37 (23.3%)	0.794
Diabetes	45 (28.3%)	48 (30.2%)	0.805
Heart attack	32 (20.1%)	29 (18.2%)	0.776
Kidney disease	16 (10.1%)	16 (10.1%)	1.000
Stroke	33 (20.8%)	31 (19.5%)	0.889
Hypertension	143 (89.9%)	131 (82.4%)	0.073

Table 2 shows the OABSS, ICIQ-SF, and VHIS scores for the two groups after PS matching. All items were adjusted to avoid significant differences.

**Table 2:** Between group differences in overactive bladder and vaginal health status after PS matching ICIQ-SF, International Consultation on Incontinence Questionnaire-Short Form; NYT, Ninjin'yoeito; OABSS, overactive bladder symptom score; VHIS, vaginal health index score; PS, propensity score

	NYT group	No-NYT group	p-value
OABSS	10.14 ±2.88	9.44 ±3.07	0.037
ICIQ-SF	12.04 ±3.75	11.06 ±4.55	0.036
VHIS	12.90 ±2.85	13.57±2.96	0.042

**Figure 2:**
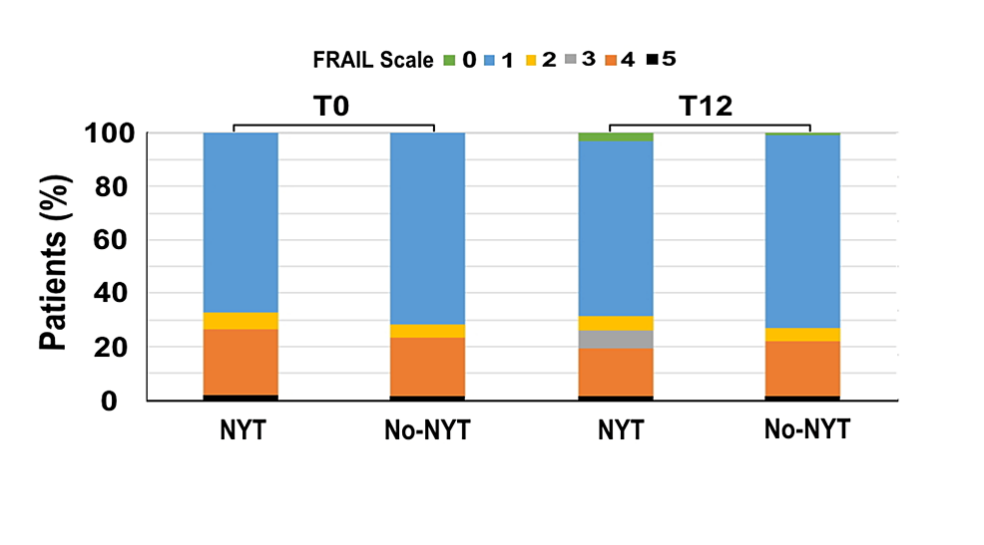
FRAIL scale scores of the two groups at T0 and T12 The vertical axis represents the percentage of patients (%) and the horizontal axis highlights the NYT and no-NYT groups at T0 and T12. Patients in the NYT group received NYT for one year, whereas those in the no-NYT group did not receive NYT. T0 represents the first visit to our hospital by patients. T12 represents the 12th month of NYT use. The green, blue, yellow, gray, orange, and black bars indicate FRAIL scale points 0, 1, 2, 3, 4, and 5, respectively. NYT, Ninjin'yoeito; FRAIL: fatigue, resistance, ambulation, illnesses, and loss of weight

Figure 2 shows the changes in the FRAIL scale scores in each group. There were no significant between group differences in FRAIL scale scores at T0 (no-NYT group: 1.73 ± 1.27; NYT group: 1.87 ± 1.35, p=0.325) and T12 (no-NYT: 1.72 ± 1.27; NYT: 1.75 ± 1.28, p=0.826). Δ_T0/T12_FRAIL scale score was significantly higher in the NYT group (0.12 ± 0.34) than in the no-NYT group (0.01 ± 0.08) (p<0.001).

**Figure 3:**
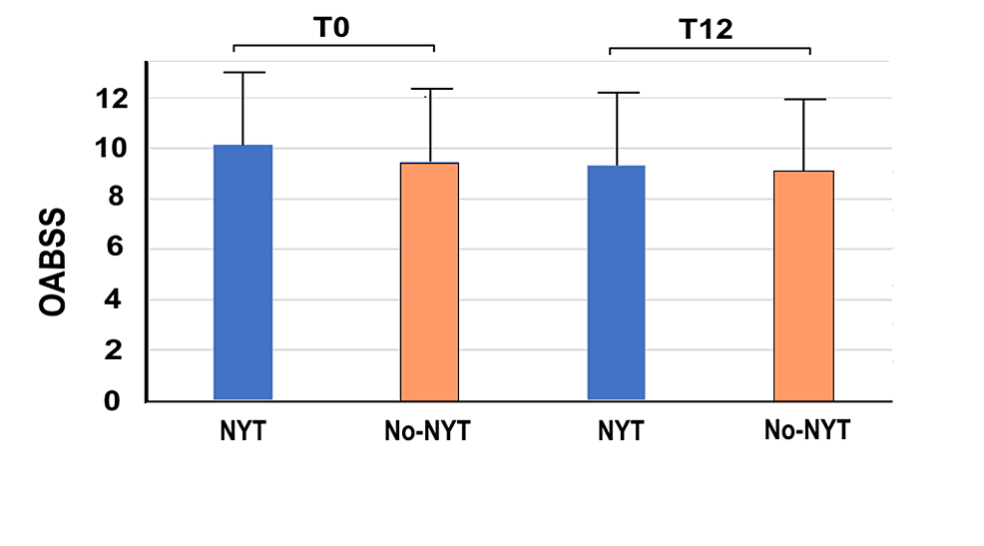
OABSS of the two groups at T0 and T12 The vertical axis represents the OABSS, whereas the horizontal axis highlights the NYT and no-NYT groups at T0 and T12. Patients in the NYT group received NYT for 1 year, whereas those in the no-NYT group did not receive NYT. T0 represents the first visit to our hospital by patients. T12 represents the 12th month of NYT use. NYT, Ninjin'yoeito; OABSS, overactive bladder symptom score

Figure 3 shows the changes in OABSS. There were no significant between group differences in OABSS at T0 (NYT group: 10.14 ± 2.88; no-NYT group: 9.44 ± 3.07, p = 0.037) and T12 (NYT group: 9.33 ± 3.24; no-NYT group: 9.08 ± 3.36, p = 0.446). From T0 to T12, five women in the NYT group and one woman in the no-NYT group went from pre-frail to no frail; moreover, two women in the NYT group and two women in the no-NYT group went from frail to pre-frail.

Δ_T0/T12_OABSS was significantly higher in the NYT group (0.89 ± 1.65) than in the no-NYT group (0.36 ± 1.14) (p=0.001), indicating that NYT use effectively improved OAB symptoms.

**Figure 4:**
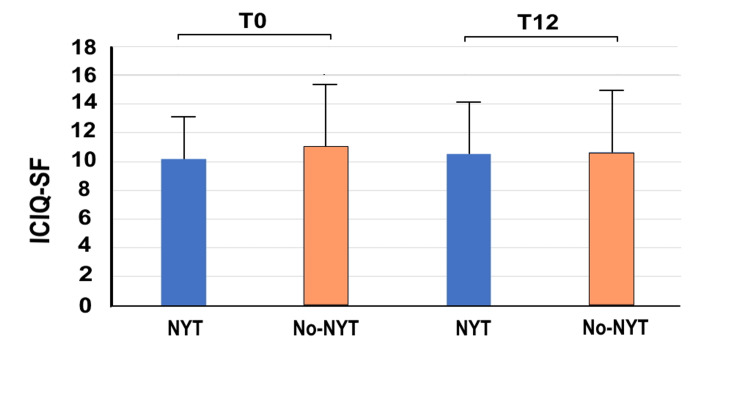
ICIQ-SF scores of the two groups at T0 and T12 The vertical axis represents the ICIQ-SF score and the horizontal axis highlights the NYT and no-NYT groups at T0 and T12. Patients in the NYT group received NYT for one year, whereas those in the no-NYT group did not receive NYT. T0 represents the first visit to our hospital by patients. T12 represents the 12th month of NYT use. NYT, Ninjin'yoeito; ICIQ-SF, International Consultation on Incontinence Questionnaire-Short Form

Figure 4 shows the ICIQ-SF scores for the two groups at T0 and T12. There were no significant between group differences in ICIQ-SF scores at T0 (NYT group: 10.14 ± 2.88; no-NYT group: 11.06 ± 4.55, p=0.037) and T12 (NYT group: 10.53 ± 3.91; no-NYT group: 10.64 ± 4.76, p=0.837). However, as with the OABSS, Δ_T0/T12_ ICIQ-SF was significantly higher in the NYT group (1.51 ±1.75) than in the no-NYT group (0.42 ±1.18) (p<0.001), indicating that NYT use improved urinary incontinence-related quality of life more effectively compared with NYT non-use.

**Figure 5:**
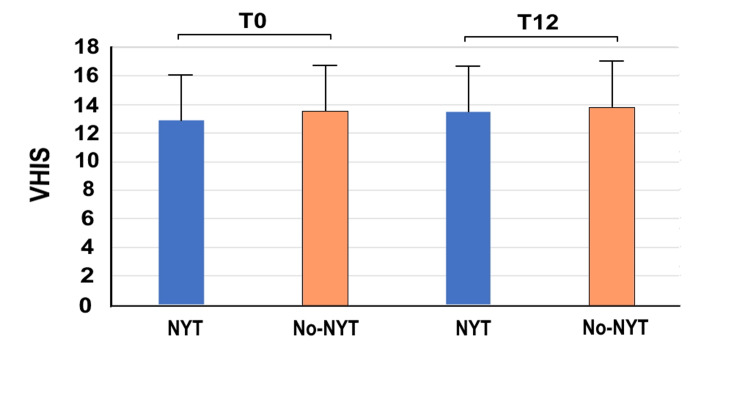
VHIS of the two groups at T0 and T12 The vertical axis represents the VHIS and the horizontal axis highlights the NYT and no-NYT groups at T0 and T12. Patients in the NYT group received NYT for one year, whereas those in the no-NYT group did not receive NYT. T0 represents the first visit to our hospital by patients. T12 represents the 12th month of NYT use. NYT, Ninjin'yoeito; VHIS: vaginal health index score

Figure 5 presents the VHIS for the two groups at T0 and T12. There were no significant between group differences at T0 (NYT group: 12.90 ± 2.85; no-NYT group: 13.57 ± 2.96, p=0.042) and T12 (NYT group: 13.48 ± 2.92; no-NYT group: 13.78 ± 3.08, p=0.381). Nevertheless, Δ_T0/T12_VHIS was significantly higher in the NYT group (0.58 ± 1.08) than in the no-NYT group (0.21 ± 0.65) (p<0.001), indicating an effective VHIS improvement with NYT use.

Table 3 shows the number and proportion of patients with vulvovaginal evoked pain. The most intense pain was induced at the left para-hymen. At T0, the proportion of patients with evoked pain decreased from 67% to 57% in the NYT group, although the difference was not significant. Moreover, the proportion of patients with evoked pain decreased from 74% to 62% in the no-NYT group at T12, although the difference was not significant. At T0, three women in each group presented with pain. At T12, two women in the NYT group showed pain improvement, whereas no women in the No-NYT group presented pain improvement.

**Table 3:** Vulvodynia swab test findings of the two groups at T0 and T12 Patients in the NYT group received NYT for one year, whereas those in the no-NYT group did not receive NYT. T0 represents the first visit to our hospital by patients. T12 represents the 12th month of NYT use. NYT, Ninjin'yoeito

	NYT group	No-NYT group	p-value
T0			
Left para-hymen	67 (42%)	74 (46%)	0.160
Right para-hymen	31 (19%)	31 (20%)	0.809
Urethra	37 (23%)	40 (25%)	0.641
Labia	10 (6.2%)	8 (5.0%)	0.702
T12			
Left para-hymen	57 (36%)	62 (38%)	0.910
Right para-hymen	29 (18%)	29 (18%)	0.807
Urethra	35 (22%)	38 (24%)	0.047
Labia	10 (6.2%)	7 (4.4%)	1.000

One (0.6%) patient presented with vaginal redness, which was the only side effect of NYT reported in this study.

We calculated the OAB medication and NYT adherence rates. Prior to PS matching, the NYT group had 409 patients, including 83 and 326 patients with and without POP, respectively. Of the NYT group patients, 92 (28.2%) discontinued their OAB medication due to missed doses or side effects. Twelve patients (3.7%) discontinued NYT for the same reason.

In contrast, the non-NYT group had 316 patients prior to PS matching, including 71 and 245 patients with and without POP, respectively. Sixty-three (25.7%) of the patients discontinued their OAB medications due to missed doses or side effects.

**Figure 6:**
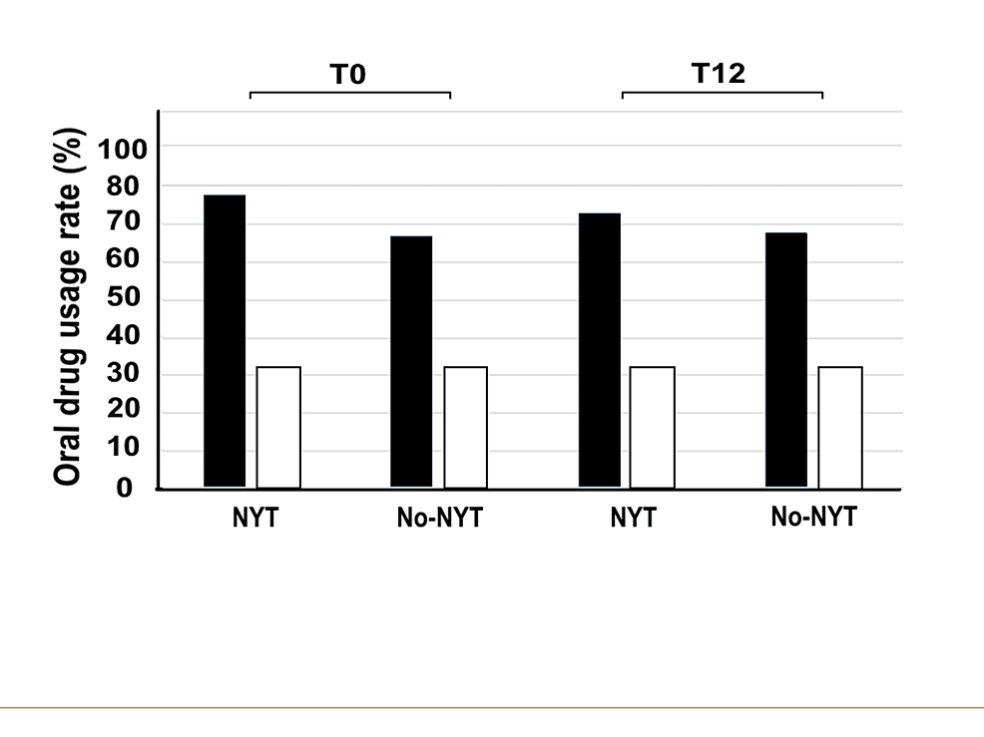
Oral OAB medication use rate in the two groups at T0 and T12 The vertical axis represents the oral drug usage rate (%) and the horizontal axis highlights the NYT and no-NYT groups at T0 and T12. Patients in the NYT group (black bar) received NYT for one year, whereas those in the no-NYT group (white bar) did not receive NYT. T0 represents the first visit to our hospital by patients. T12 represents the 12th month of NYT use. NYT, Ninjin'yoeito; OAB, overactive bladder

Figure 6 shows OAB medication use in the two groups after PS matching. At T0, 32% and 78% of patients in the NYT group used β3 adrenoceptor agonists and antimuscarinic drugs, respectively. Similarly, 32% and 67% of patients in the no-NYT group used these drugs, respectively. In T12, 32% and 73% of patients in the NYT group used β3 adrenoceptor agonists and antimuscarinic drugs, respectively. Hence, there was a 5% decrease in the proportion of antimuscarinic drug users. However, 32% and 68% of patients in the no-NYT group used these drugs, respectively, indicating a 1% increase in the proportion of antimuscarinic drug users. All patients were on oral medications.

## Discussion

Our study revealed significant improvements in various parameters associated with frailty and genitourinary symptoms in the NYT group compared to the no-NYT group. Specifically, the ratios expressed as Δ_T0/T12_FRAIL scale score, Δ_T0/T12_OABSS, Δ_T0/T12_ICIQ-SF, and Δ_T0/T12_VHIS demonstrated notable positive effects of NYT. These findings highlight the potential of NYT in improving frailty-related aspects, including sarcopenia, grasping power, and fatigue, as supported by the relevant published literature [16].

In our study, after PS matching, there was no significant difference in the FRAIL scale between the NYT group and the no-NYT group. Additionally, factors related to FRAIL, such as angina, arthritis, asthma, cancer, chronic lung disease, congestive heart failure, diabetes, heart attack, kidney disease, and stroke, did not differ between the two groups. However, the Δ_T0/T12_FRAIL scale score significantly improved in the NYT group compared to the no-NYT group. This effect of NYT use was predictable based on previous study findings mentioned below. The effect of NYT use can be predicted based on previous research that has reported various beneficial effects of NYT. NYT is composed of 12 crude drugs, including *Rehmannia* root, Japanese angelica root, *Atractylodes* rhizome, peony root, cinnamon bark, astragalus root, poria sclerotium, *Citrus unshiu* peel, polygala root, *Glycyrrhiza*, and schisandra fruit, which have been the subject of previous studies that demonstrated its efficacy [13,17-24].

Uto et al. reported that glycyrrhizic acid in licorice has anti-inflammatory properties [13], and that peoniflorin relieves muscle pain [13]. Ginseng has been shown to have anti-fatigue and antidepressant effects [13]. *C. unshiu* peels inhibit atrophy and nerve cell apoptosis [13]. The components narirutin and hesperidin improve cognitive function. Hesperidin restores ghrelin secretion [13]. Ito et al. investigated the efficacy of NYT use in the treatment of subjective fatigue resulting from lenalidomide and dexamethasone treatment [23]. Kudoh et al. reported that the use of NYT may potentially contribute to the improvement of cognitive function and depressive symptoms in patients with Alzheimer's disease [24].

In our study, Δ_T0/T12_OABSS was significantly higher in the NYT group (0.89 ± 1.65) compared to the no-NYT group (0.36 ± 1.14) (p = 0.001), indicating a significant improvement in OAB with NYT use. Additionally, Δ_T0/T12_ICIQ-SF score was significantly higher in the NYT group (1.51 ± 1.75) than in the no-NYT group (0.42 ± 1.18) (p < 0.001). The ICIQ-SF is a test that evaluates the subjective quality of life improvement related to urinary incontinence. Patients with frailty should be able to perceive the improvement in quality of life subjectively. In the NYT group, the ICIQ-SF score worsened from 10.14 ± 2.88 (T0) to 10.53 ± 3.91 (T12), although Δ_T0/T12_ICIQ-SF still showed improvement. This suggests a synergistic relationship between improved depression symptoms and OAB improvement in the NYT group, as reported in a previous study [24].

There are differing opinions among researchers regarding the relationship between frailty and OAB. In previous studies, Yoshida et al. found a positive association between frailty and the occurrence of OAB in older individuals [5]. Out of 2,953 participants (65% men) aged 65 years or older, 150 (5.1%) had frail OAB, 416 (14.1%) had non-frail OAB, 287 (9.7%) had frail non-OAB, and 2,100 (71.1%) had non-frail non-OAB. Among the 289 patients with severe/moderate OAB, 84 were frail and 97 were pre-frail. Among the 277 patients with mild OAB, 66 were frail and 102 were pre-frail. However, Shaw et al. conducted a review and reported limited evidence of a direct link between frailty and OAB, although urinary urgency may serve as a precursor to frailty in older adults [25].

Our study was not a cross-sectional study evaluating differences in VHIS or vulvodynia swab test findings between patients with and without frailty. However, at T0, the VHIS values were 12.90 ± 2.85 and 13.57 ± 2.96 for the NYT and no-NYT groups, respectively, which were below the standard value of 15. In the vulvodynia swab test, the left para-hymen evoked pain was significantly high in both the no-NYT (67 patients, 42%) and NYT (74 patients, 46%) groups. Nevertheless, there was no difference between the two groups in terms of vulvodynia occurrence, probably because the pain was not quantified, and therefore could not be analyzed.

To the best of our knowledge, no research reports are available on the association between frailty and conditions such as "GSM," "vulvodynia," "VHIS," and "atrophic vaginosis". However, there is a close connection between the urethra and vaginal labia, which may explain the occurrence of GSM. For instance, vulvodynia is prevalent in patients with interstitial cystitis, which is considered a bladder disease [26]. Additionally, breast cancer (BC) survivors have a higher incidence of vulvodynia [27]. A meta-analysis of 24 studies involving 13,510 BC patients indicated a relatively high prevalence of frailty in BC patients, and BC treatment conditions may increase the risk of frailty [28].

In our study, antimuscarinic drug use decreased by 5% in the NYT group without significant side effects. These findings suggest that NYT use may improve urinary and genital symptoms, as well as frailty, addressing polypharmacy in frail patients.

Johnson et al. evaluated OAB medication usage in 2,527 patients in the United States who were prescribed either mirabegron (21%) or an antimuscarinic drug (79%) [29]. Among them, 1,032 used antimuscarinic drugs (median age: 81 years, 62% female) and 516 used mirabegron (median age: 82 years, 64% female). Antimuscarinic drug users had lower compliance (33.8% vs. 39.1%) and a shorter duration of use (90 days vs. 103 days). The study also indicated higher healthcare costs for frail OAB patients compared to the general OAB population.

The relevance of our findings extends beyond genitourinary symptoms and frailty. Sarcopenia has emerged as a significant concern in developed countries, given the impact of aging populations. Building upon the revised European consensus on the definition and diagnosis of sarcopenia [30], our study provides novel insights into the multifaceted benefits of NYT, including its potential to improve genitourinary symptoms associated with frailty, thus addressing important issues related to aging, polypharmacy, and quality of life.

Our study has some limitations. First, there was no placebo control group in this study, and although the NYT and no-NYT groups were significantly different after PS matching, a randomized controlled trial is necessary to provide more accurate results. Second, it is important to note that while the FRAIL questionnaire has been translated into Japanese and is widely used, there is currently no published literature validating the Japanese version. This lack of validation may introduce some uncertainty in the interpretation of the frailty assessment. Additionally, it is important to acknowledge that Kampo medicine is routinely practiced in Japan, and patients may have varying attitudes and impressions towards it, potentially introducing a bias. In our study, patients who had a favorable impression of Kampo medicine were administered NYT with PFMT, while those who did not have a positive impression underwent PFMT without NYT administration.

## Conclusions

In this study of frail and pre-frail patients, urinary and genital symptoms were observed. The study investigated the use of NYT, which is commonly used in Japan for frailty treatment. The NYT group showed improved frailty scores, suggesting potential for enhanced activity levels. Additionally, improvements in OABSS, ICIQ-SF, VHIS, and vulvodynia swab test were seen in the NYT group, indicating potential relief of urinary and genital symptoms. Furthermore, the NYT group had fewer antimuscarinic drug users. These findings suggest the potential of NYT in addressing urinary system issues.

To strengthen the evidence, a more rigorous placebo-controlled trial design, objective outcome measures, studying biological mechanisms, longer follow-up, and replication in more diverse populations would be beneficial.
